# Plant 45S rDNA Clusters Are Fragile Sites and Their Instability Is Associated with Epigenetic Alterations

**DOI:** 10.1371/journal.pone.0035139

**Published:** 2012-04-11

**Authors:** Min Huang, Hui Li, Lu Zhang, Fei Gao, Pu Wang, Yong Hu, Shihan Yan, Lin Zhao, Qi Zhang, Junjun Tan, Xincheng Liu, Shibin He, Lijia Li

**Affiliations:** State Key Laboratory of Hybrid Rice, College of Life Sciences, Wuhan University, Wuhan, China; Texas A&M University, United States of America

## Abstract

Our previous study demonstrated that 45S ribosomal DNA (45S rDNA) clusters were chromosome fragile sites expressed spontaneously in *Lolium*. In this study, fragile phenotypes of 45S rDNA were observed under aphidicolin (APH) incubation in several plant species. Further actinomycin D (ActD) treatment showed that transcriptional stress might interfere with chromatin packaging, resulting in 45S rDNA fragile expression. These data identified 45S rDNA sites as replication-dependent as well as transcription-dependent fragile sites in plants. In the presence of ActD, a dramatic switch to an open chromatin conformation and accumulated incomplete 5′ end of the external transcribed spacer (5′ETS) transcripts were observed, accompanied by decreased DNA methylation, decreased levels of histone H3, and increased histone acetylation and levels of H3K4me2, suggesting that these epigenetic alterations are associated with failure of 45S rDNA condensation. Furthermore, the finding that γ-H2AX was accumulated at 45S rDNA sites following ActD treatment suggested that the DNA damage signaling pathway was associated with the appearance of 45S rDNA fragile phenotypes. Our data provide a link between 45S rDNA transcription and chromatin-packaging defects and open the door for further identifying the molecular mechanism involved.

## Introduction

Eukaryotic genomes contain hundreds of tandemly arranged 45S ribosomal RNA (rRNA) genes (45S rDNAs) which are transcribed by RNA polymerase I to generate 18S, 5.8S and 28S rRNAs. The 45S rDNA region shows some typical characteristics of genetic instability, which often results in variable copy number and distribution among closely related species and even intraspecies. The studies revealed that the whole 45S rDNA repeats in *Allium* and subgenus *Mus* chromosomes could free shift from one locus to another, suggesting that 45S rDNA might serve as a transposable element [1], [2]. In *Neurospora*, nucleolus organized region (NOR) breakage caused large terminal deletions and such 45S rDNA termini were healed by the addition of telomeric repeats to prevent terminus fusion [3]. In *Lolium rigidum*, *in vivo* 45S rDNA breakage led to chromosome rearrangement and varied position and number of 45S rDNA sites [4]. It has been reported that 45S rDNA clusters are preferred sites for gene rearrangements and chromosome breakage-fusion-bridge cycles in telomerase-deficient *Arabidopsis* [5]. The 45S rDNA instability might be related to its transcriptional ability [3]. This hypothesis was tested using a transcription inhibitor actinomycin D (ActD). ActD is an anti-tumor antibiotic and forms a stable complex with GpC-rich nucleotide clusters particularly in transcriptionally active regions, thus inhibiting the elongation of growing RNA chains [6]. For example, the NORs seemed to be the most likely regions of stretching and breaks in indian muntjac cells following treatment with ActD [7]. Recent work has demonstrated that 45S rDNA regions as the chromosome fragile sites are spontaneously expressed *in vitro* on metaphase chromosomes in *Lolium* [8] and this fragility is likely to account for the observed genetic instability.

Fragile sites are large and highly sensitive regions that are inclined to form abnormal poor–staining lesions (gaps, constrictions or breaks) in metaphase chromosomes [9], [10]. Currently, the well characterized fragile sites are thought to be caused by DNA replication-dependent defects, which therefore can be induced and enhanced when cells are partially inhibited by replication stress that retards DNA replication fork progression at fragile sites, such as exposure to the DNA polymerase inhibitor aphidicolin (APH) [11], [12]. The alteration of DNA sequence motifs and epigenetic modifications interfere indirectly or directly with the higher-order chromatin packing, thus may be involved in the appearance of the fragile sites [13], [14]. Two replication checkpoint proteins (ATR and BRCA1) were found to participate in maintaining the stability of fragile sites [15], [16]. In addition, adenovirus type 12 can induce the appearance of fragile phenotypes of the RNU2 locus in human [17].

In this study, our data identify plant 45S rDNA regions as fragile sites derived from replication-associated as well as transcription-dependent defects, and establish that the ActD-induced transcription stress gives rise to a dramatic switch to an open chromatin conformation and an accumulation of incomplete 5′ETS, accompanied by decreased DNA methylation, decreased levels of histone H3, and increased histone acetylation and levels of H3K4me2. The epigenetic alterations might result in the inability of chromatin fibers to fold into higher-order configurations, which may contribute to the appearance of 45S rDNA fragile phenotypes. Furthermore, γ-H2AX accumulation at 45S rDNA sites suggests that ActD-mediated DNA damage is involved in the transcription-dependent fragile expression process.

## Results

### Aphidicolin treatment results in 45S rDNA fragility in plants

**Figure 1:**
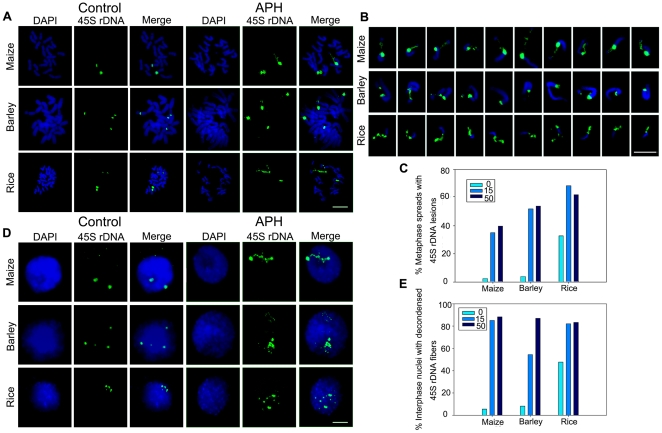
APH induces 45S rDNA fragility in maize, barley and rice. (A) Metaphase chromosome spreads revealed aberrant 45S rDNA phenotypes induced by APH. The 45S rDNA FISH signals appeared as compact spots on chromosomes in untreated plants. APH-induced 45S rDNA lesions were localized randomly on either or both of a pair of homologous chromosomes from maize, barley, and rice after treatment with 15 µg/ml APH. Bar = 5 µm. (B) Examples of types of lesions observed at 45S rDNA sites after treatment with APH for 48 h. Bar = 5 µm. (C) Percentages of metaphase spreads with 45S rDNA lesions after treatment without or with 15 µg/ml and 50 µg/ml APH, respectively. Number of spreads evaluated in each group was 300. (D) APH treatment caused aberrant 45S rDNA signal patterns in nuclei. FISH with 45S rDNA probes showed spot signals in normal plants and fiber-like threads unraveled from compacted states in nuclei treated with 15 µg/ml APH. Bar = 10 µm. (E) Percentages of interphase nuclei with decondensed 45S rDNA fibers after treatment without or with 15 µg/ml and 50 µg/ml APH, respectively. Number of evaluated nuclei in each group was 500.

Chromosome fragile sites represent regions which are inclined to form poor–staining constrictions, breaks or gaps in metaphase chromosomes when DNA replication is partially inhibited [18]. The finding that 45S rDNA regions are spontaneously-formed fragile sites in metaphase chromosomes in *Lolium* species [8] prompted us to investigate whether the 45S rDNA repeats in other plant species such as maize, barley and rice displayed fragility after treatment with DNA polymerase inhibitor aphidicolin (APH). In pilot experiments we treated seedlings with a range of concentrations of APH, and found that 15 µg/ml and 50 µg/ml of APH treatment caused a significant increase of 45S rDNA lesions but allowed near normal growth and development. However, a fraction of seedlings ceased growth when the concentration reached 100 µg/ml. Thus, 15 µg/ml and 50 µg/ml of APH were selected for this study. The 45S rDNA FISH signals are normally represented as dense dots in both sister chromatids on metaphase chromosomes (Figure 1A). Inhibition of DNA synthesis by APH caused lesions or aberrances of 45S rDNA regions in a large fraction of chromosome spreads in all of the treated plants (Figure 1A and 1B). In some cases, the signals looked like beads on a string emanating from a big dot signal as if the rDNA chromatin failed to fold completely. The APH-induced defects often occurred at or close to a 45S rDNA terminus, thus giving rise to spatially separated ends containing no or only very weak 45S rDNA signals. The FISH signal of rDNA lesions was heterogeneous, consistent with the observation in *Lolium* [8], and occurred randomly on either or both of a pair of homologous chromosomes. The fraction of metaphase spreads with 45S rDNA lesions was different among analyzed species after treatment with 15 µg/ml APH for 48 h (34.9% for maize, 51.9% for barley and 68.4% for rice) (Figure 1C). The incidence of lesions was similar when the concentration of APH was 50 µg/ml (Figure 1C). In normal interphase nuclei, a majority of 45S rRNA genes appeared to be compacted into heterochromatic chromocenters and FISH signals appeared as compacted spots. In the presence of APH, this well-organized structure was disturbed (Figure 1D). The “beads-on-a-string” fibers appeared to be unraveled from their normal compact states and were spread throughout the nucleoplasm (Figure 1D). The fraction of the nuclei with fiber-like threads was greater than 50% in all tested plants when the APH was applied at a concentration of 15 µg/ml for 48 h (and was >80% at 50 µg/ml) (Figure 1E). Further, dual-color FISH with 45S rDNA and centromere probes in maize showed that lesions were located at the NOR, with no visible damage on the primary constriction (Figure S1A). Consistent with the observation on metaphase chromosomes, heterochromatic centromeres in nuclei remained compacted following APH treatment (Figure S1B). We also analyzed the effect of APH on maize heterochromatic knobs which possess distinctive cytological appearances and are capable of inducing neocentric activity [19]. Similarly, 45S rDNA lesions were observed, but all of the knob signals remained as intact blocks of heterochromatin in chromosomes and nuclei in cells treated with APH (Figure S1C and S1D). These observations showed that chromosome lesions were associated with 45S rDNA regions but not centromeres or knobs, possibly reflecting stalled replication of rDNA due to APH inhibition. We conclude that the 45S rDNA cluster is a fragile site in plant genome that may be caused by defects in DNA replication and the observed extended rDNA chromatin is a consequence of fragile rDNA repeats.

### Fragile phenotypes are related to transcription activity revealed by AgNOR staining

**Figure 2:**
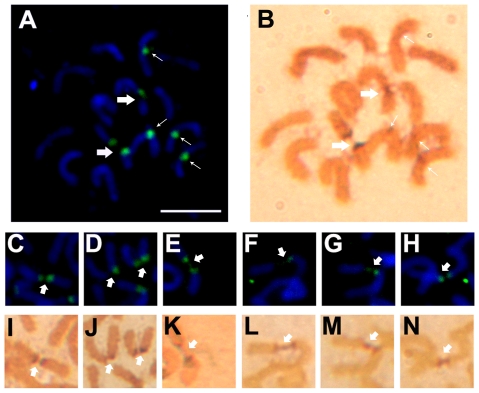
Co-localization of Ag-NOR proteins and 45S rDNA lesions in untreated ryegrass metaphase chromosomes. (A) FISH mapping showed two 45S rDNA lesions (thick arrow) and four intact 45S rDNA sites (thin arrow). (B) Intense staining of Ag-NOR proteins co-localized at the same lesion site shown in figure A (thick arrow). In contrast, there was no signal or only very weak signals detected at intact 45S rDNA sites (thin arrow). (C–H) Examples of different cytological appearances of 45S rDNA lesions (thick arrow). (I–N) A one-to-one comparison was made between silver-staining signals and 45S rDNA lesions shown in figure C–H (thick arrow). Bar = 10 µm.

Our previous study proposed that the failure of chromatin folding at 45S rDNA regions contributed to the occurrence of chromosome lesions in ryegrass [20]. It has been demonstrated that decondensed chromatin fibers form an open conformation for highly efficient transcriptional events [21]. AgNOR staining was performed to determine whether transcription activity was correlated to the fragile phenotype of 45S rDNAs in ryegrass. AgNOR proteins have an affinity for reductive silver under acidic conditions and remain associated with the NOR during mitosis, which allow them to be good markers to distinguish actively transcribed rRNA genes from inactive ones [22], [23]. Comparison of 45S rDNA hybridization signals and AgNOR staining signals on the same metaphase chromosome spread indicated that specific strong AgNOR staining signals co-localized with broken or decondensed 45S rDNA sites (Figure 2A–2N). In contrast, there was no signal or only very weak signals detected at intact 45S rDNA sites (Figure 2B). The differential susceptibility of the 45S rDNA loci to AgNOR staining revealed that transcription by Pol I might prevent the condensation of DNA during metaphase, ultimately resulting in fragile 45S rDNA sites.

### Fragile 45S rDNA sites are induced by actinomycin D in treated plants

**Figure 3:**
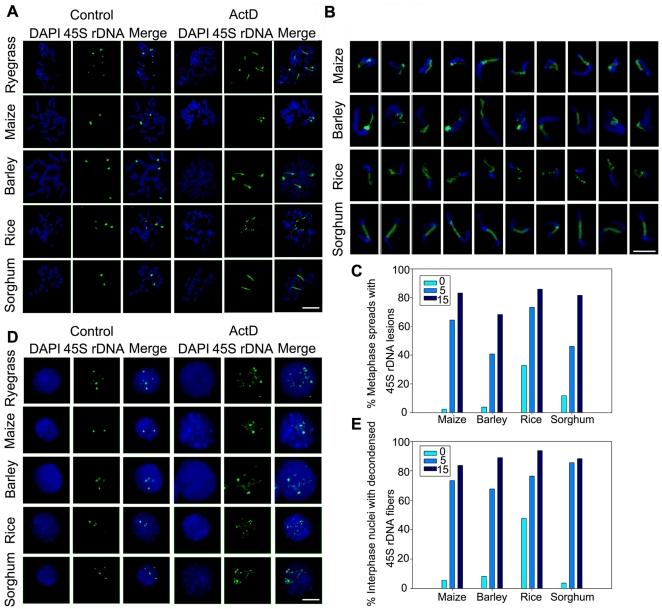
ActD induces 45S rDNA fragility in ryegrass, maize, barley, rice and sorghum revealed by FISH. (A) Metaphase chromosome spreads revealed aberrant 45S rDNA phenotypes induced by ActD. The 45S rDNA FISH signal was the dense spot on chromosomes in untreated plants. In contrast, ActD-treated spreads exhibited highly stretched strands of rDNA signals or breaks on chromosomes after treatment with 15 µg/ml ActD. Bar = 5 µm. (B) Examples of fragile 45S rDNA phenotypes Bar = 5 µm. (C) Percentages of metaphase chromosome spreads with 45S rDNA lesions after treatment without or with 5 µg/ml and 15 µg/ml ActD, respectively. Number of evaluated spreads in each group was 300. (D) ActD treatment caused aberrant 45S rDNA signal patterns in nuclei. The interphase nuclei contained compact spot signals in normal plants whereas a mass of “beads-on-a-string” fibers were observed throughout the nucleoplasm in interphase nuclei after treatment with 15 µg/ml ActD. Bar = 10 µm. (E) Percentages of interphase nuclei with decondensed 45S rDNA fiber signals after treatment without or with 5 µg/ml and 15 µg/ml ActD, respectively. Number of evaluated nuclei in each group was 500.

We next investigated whether transcription activity is related to 45S rDNA fragility in plants by using a transcription inhibitor, actinomycin D (ActD), to block rRNA gene transcription. It was reported that the low concentration of ActD (50 or 100 µg/ml) specifically inhibited rRNA synthesis in *Vicia* [24]. After testing a series of concentrations of ActD (0.5 µg/ml, 5 µg/ml, 15 µg/ml and 50 µg/ml), 5 µg/ml and 15 µg/ml ActD were chosen because they induced 45S rDNA decondensation but had little effect on seedling growth. FISH on chromosomes using the 45S rDNA as a probe revealed that the NORs seemed to be more decondensed and stretched in ryegrass under conditions of transcriptional inhibition (Figure 3A). This phenomenon was similar to what was observed in electron micrographs showing that ActD treatment induced chromosome and chromatid breaks and other aberrations occurred exclusively on NORs in Indian muntjac cells [7]. Similarly, these metaphase chromosomal abnormalities (aberrations) at NORs induced by ActD were frequently observed in maize, barley, rice and sorghum, and FISH results showed distinct fragile appearances, ranging from spatially separated ends linked with no or only a few thin rDNA fiber threads to highly stretched strands of 45S rDNA signals (Figure 3A and 3B). The cytological phenotypes of 45S rDNA fragile site were gaps or breakage when the green signal layer was removed, resembling those of common fragile sites reported in human metaphase chromosomes [25]. Different plant species showed different sensitivity to ActD, and thus the incidence rates varied among plants (64.4% for maize, 40.8% for barley, 73.3% for rice and 46.2% for sorghum) after treatment with 5 µg/ml ActD for 48 h (Figure 3C). The frequency of chromosomal lesions at 45S rDNA sites increased significantly as the ActD concentration was increased (Figure 3C). In most of the chromosome spreads of maize and sorghum, the chromosome breakage occurred at both of the homologous 45S rDNA sites. In rice, there are two pairs of 45S rDNA sites located at the ends of chromosomes 9 and 10 respectively [26]. The major 45S rDNA sites on chromosome 9 seemed to be more susceptible to stretching than weaker sites on chromosome 10. However, the number of lesions varied from cells to cells and it appeared that lesions appeared randomly and heterogenously at every 45S rDNA repeat unit in barley and ryegrass, consistent with spontaneous lesions in ryegrass [8]. ActD also resulted in 45S rDNA decondensation in interphase nuclei, which exhibited as fiber-like thread signals after FISH (Figure 3D). The frequency of such nuclei surpassed 65% in all tested plants, when the ActD was applied at the concentration of 5 µg/ml for 48 h (and was >80% at 15 µg/ml) (Figure 3E). Furthermore, ActD exerted no obvious effect on centromeres and knobs in maize (Figure S2). Taken together, these results demonstrate that 45S rDNAs are regions of fragility in plant chromosomes, and decondensation or breakage at NORs is induced after exposure to both ActD and APH, suggesting that this fragility is transcription-dependent as well as replication-dependent. We next concentrated on exploring the molecular mechanism concerning the impact of ActD exposure on the appearance of fragile phenotypes at the 45S rDNA in maize.

### Effects of ActD on rRNA transcription are revealed by AgNOR staining and quantitative analysis

**Figure 4:**
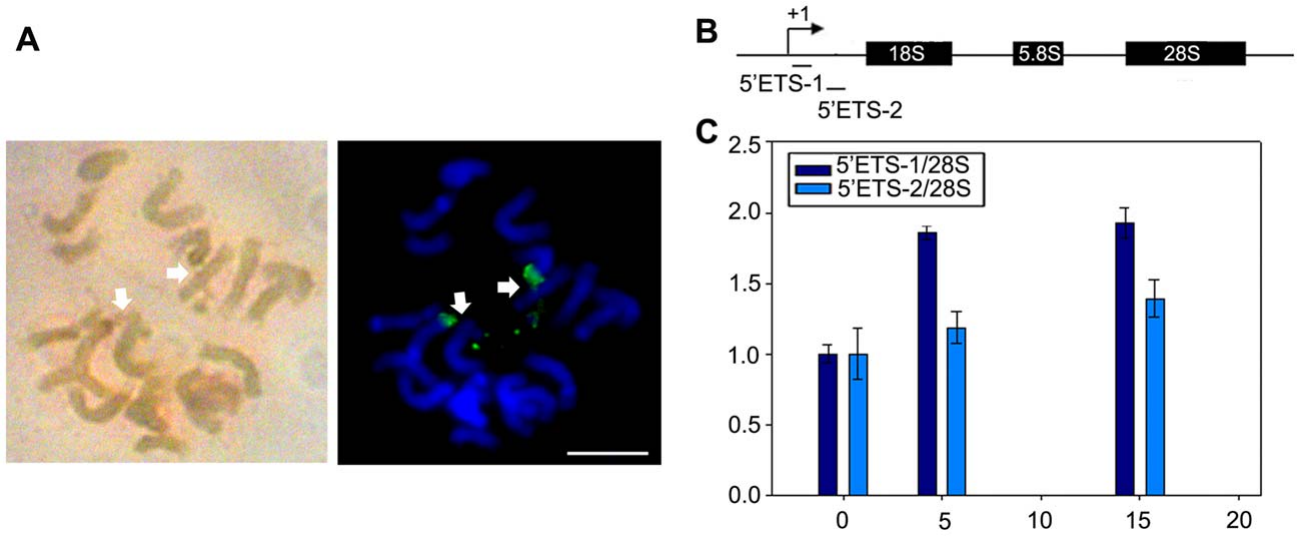
Effects of ActD on rRNA transcription in maize. (A) Co-localization of Ag-NOR proteins (left) and NOR site extension (right) in maize metaphase chromosomes after treatment with 15 µg/ml ActD. Arrows indicated extended NORs. Bar = 5 µm. (B) Schematic representation of the maize rRNA genes and the primers for quantitative analysis. (C) Quantitative real-time PCR analysis of 5′ETS transcripts in maize after treatment without or with 5 µg/ml and 15 µg/ml ActD, respectively. ActD treatment accumulated incomplete 5′ETS transcripts in maize. The y-axis indicated relative expression values and the x-axis indicated the various concentrations of ActD. Expression values were normalized to the levels of 28S rRNA. Relative expression ratio of each sample was compared to the maize root without ActD treatment which was assigned to the value of 1. Each experiment was repeated three times and the average value was shown with the SD.

To demonstrate the link between rRNA transcription and NOR site extension, the AgNOR staining experiment was performed. The AgNOR staining signals appeared as two spots linked by fiber-like signals which co-localized with the decondensed NOR regions from cells treated with 15 µg/ml ActD (Figure 4A). These results raised the possibility that the rRNA transcript was still synthesized in the presence of ActD. Quantitative real-time PCR was further applied to analyze the effect of ActD treatment on de novo synthesis of ribosomal RNA precursors (pre-rRNA) in maize. Primers were specific for 5′ end of the external transcribed spacer (5′ETS-1 and 5′ETS-2, Table 1). To exclude the contamination of mature rRNAs, the amount of 28S rRNAs was included as internal control. Obviously, the amount of 5′ETS-1 and 5′ETS-2 transcripts was increased under the pressure of both 5 ug/ml ActD and 15 ug/ml ActD (Figure 4C). It raised the possibility that the transcription pressure caused more 45S rDNA repeat units to initiate transcription. At a certain concentration of ActD, the expression level of 5′ETS-2 transcripts was lower than that of 5′ETS-1 transcripts (Figure 4C), suggesting that the accumulated 5′ ETS transcripts were incomplete since the elongation of Pol I was partly inhibited by the treatment of ActD. It was concordant with the early results that the low doses of ActD could stimulate rRNA gene transcription initiation events *in vivo* although Pol I elongation was strongly inhibited [27].

**Table 1:** Primers used for quantitative analysis and DNA methylation analysis.

Amplicon	Forward primer	Reverse primer
5′ ETS-1	TGGCCCGTTGCTTGATGCGT	ACGGCCGAGCCATCCAAAGC
5′ ETS-2	ACCCTGTGCCTGCTGCGTTG	GGGGTGAGCTCGACGAAGGC
Prom-1	TTTTTGATTTTGAGTAAAAA	ACCAAACAACCAACAAC
Prom-2	TTTCGGCCACGACCGCGAAA	GGTGCACCCGAACTTCCACGT
18S	CGGGCGCGTTAGTGTCTGGT	AATTACCGCGGCTGCTGGCA
ITS1	AACGAGTCACCCGTGCCGC	GGGGCGCCGTGGGTTCTTT
5.8S	TCGGCTCTCGCATCGATGAAGA	CAGAAGGCTTCGGGCGCAACT
ITS2	CGAGGTGGGCCGAAGCAGG	GGGACGCTGCACCGAGAACA
28S	GCCGGAAGAGCACCGCACAT	ACGACCGGGCGTGGATGGTA

### Fragmented nucleoli are induced by ActD treatment in maize

**Figure 5:**
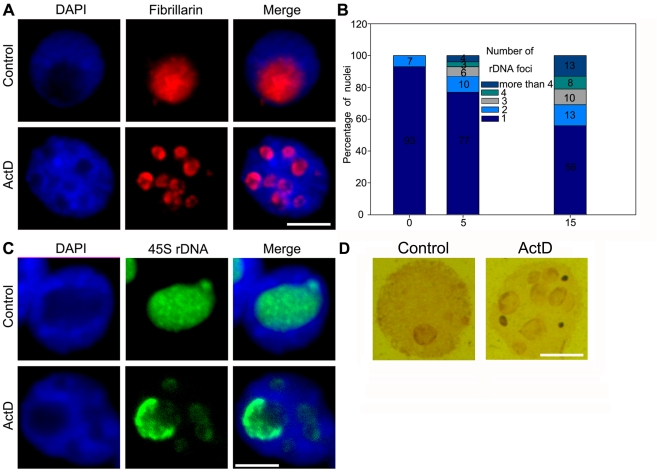
ActD causes fragmented nucleoli in Maize. (A) Fragmented nucleoli were detected by indirect immunofluorescence staining with an antibody against fibrillarin. The upper panel showed only one brightly-stained nucleolus in an untreated nucleus. The lower panel showed eight stained domains in a nucleus treated with 15 µg/ml ActD. Bar = 10 µm. (B) Percentages of interphase nuclei with fragmented nucleoli after treatment without or with 5 µg/ml and 15 µg/ml ActD, respectively. Number of evaluated nuclei in each group was 500. (C) Fragmented nucleoli were detected by FISH with 45S rDNA probes for detection of rDNAs and rRNAs in interphase nuclei. The upper FISH image showed only one domain containing hybridization signals in an untreated nucleus. The lower FISH image showed five domains containing hybridization signals in a nucleus treated with 15 µg/ml ActD. Bar = 10 µm. (D) AgNOR staining signals showed only a nucleolus in an untreated nucleus but several fragmented nucleoli in an nucleus treated with 15 µg/ml ActD. Bar = 10 µm.

In interphase nuclei, active rDNA transcription generates a nucleolus, where rRNA maturation and its assembly with specific nucleolar proteins such as fibrillarin, nucleophosmin and nucleolin occur [28]. Therefore, we examined whether nucleolar organization was altered following ActD treatment. After inhibition of rRNA synthesis in maize, indirect immunofluorescence staining for fibrillarin, a nucleolar protein participating in pre-rRNA processing, revealed numerous stained signal sites in nuclei, indicating that multiple nucleoli were present (Figure 5A and 5B). In contrast, the control nuclei contained only one or two brightly-stained domains (Figure 5A and 5B). FISH with 45S rDNA probes was applied for detection of rDNAs and rRNAs, confirming that the number of domains containing 45S rDNA hybridization signals varied from one to ten in response to ActD stress (Figure 5C). The fragmented nucleoli were further observed by AgNOR staining signals (Figure 5D). It seemed that transcription inhibition by ActD caused many of the rRNA genes to disperse throughout the nucleoplasm, which contributed to the formation of multiple nucleoli. In summary, the continued transcription of rRNA is essential to maintain the organization of the nucleolus, but when this process is disrupted, the redistribution of related components and nucleolar reorganization emerge immediately.

### ActD treatment causes site-specific hypomethylation within the 45S rDNA promoter in maize

**Figure 6:**
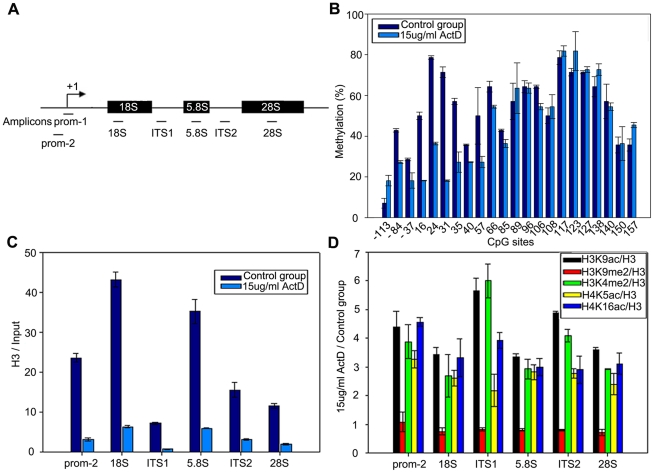
Analysis of DNA methylation and histone modifications of 45S rDNA regions in maize. (A) Schematic representation of the maize rRNA genes and the positions of analyzed amplicons. (B) ActD treatment induced site-specific hypomethylation within the 45S rDNA promoter. The y-axis indicated the ratio of the clones with methylation sites and the x-axis indicated positions of CpG dinucleotides from −113 to +157. (C) ChIP analysis showed that the total level of histone H3 within 45S rDNA regions was decreased after treatment with 15 µg/ml ActD. DNA associated with histone H3 was immunoprecipitated with the anti-H3 antibody and primers specific for different region of 45S rDNAs were used to amplify DNA for quantitative real-time PCR. The y-axis values were the relative quantities of DNA and the x-axis indicated different regions of 45S rDNAs. Each experiment was repeated three times and the average value was shown with the SD. (D) ChIP analysis of levels of H3K9ac, H3K9me2, H3K4me2, H4K5ac and H4K16ac within 45S rDNA regions in maize after treatment without or with 15 µg/ml ActD. DNA associated with different histone modifications was immunoprecipitated with the related antibody and primers specific for different regions of the 45S rDNA were used to amplify DNA for quantitative real-time PCR. The y-axis indicated the ratio of the relative quantities of DNA in maize with ActD treatment to the relative quantities of DNA in maize without ActD treatment. Relative values were normalized to those of the total H3.The x-axis indicated different regions of 45S rDNAs. Each experiment was repeated three times and the average value was shown with the SD.

The plant rRNA genes are in a tandem repetitive cluster that contains 18S, 5.8S, 28S tracts, internal transcribed spacers (ITS) and external transcribed spacers (ETS) (Figure 6A). DNA methylation is thought to play an important role in regulating the rate of rRNA gene transcription and maintaining the morphological organization of rDNA chromatin and nucleoli [29], [30]. Thus, bisulfite genomic sequencing was applied to determine the methylation modification of the 22 CpG sites within a 307 bp region throughout the 45S rDNA promoter. The rRNA gene from analyzed clones in ActD-induced cells showed significant site-specific hypomethylation at five CpG dinucleotides (positions 16, 24, 31, 35 and 57) (Figure 6B). For other CpG residues, there were also extensive alterations in DNA methylation, ranging from inconspicuous loss of CpG methylation to a slight hypermethylation (Figure 6B). These results suggest that rRNA gene decondensation caused by treatment with ActD may depend at least in part on DNA hypomethylation.

### Remarkable alterations of histone modifications are induced by ActD treatment in maize

Reversible histone modifications are believed to be epigenetic switches responsible for the rRNA genes on/off state. Thus, to ascertain whether the histone modifications were involved in regulation of 45S rDNA chromatin structures, we performed chromatin immunoprecipitation (ChIP) experiments with antibodies specific to H3, H3K9ac, H3K9me2, H3K4me2, H4K5ac and H4K16ac. Apparently, total levels of histone H3 were reduced to a lower density ranging from nearly 11.1% to 20.5% in all analyzed regions in the presence of ActD (Figure 6C). These changes were consistent with early conclusions that decondensed rRNA genes appeared to suffer a pronounced reduction in core histone molecules [31], [32]. Compared to H3, the levels of H3K9ac and H3K4me2 increased significantly at every amplicon that was analyzed, especially at the region of ITS1, and similar results were obtained using antibodies against H4K5ac and H4K16ac (Figure 6D). In contrast, the density of H3K9me2 was slightly decreased (Figure 6D). As the well-established histone code hypothesis that demonstrated that chromatin decondensation is characterized by histone hyperacetylation and increased H3K4me2, whereas H3K9me2 was an epigenetic indicator of repressed chromatin states, our observations show that histone modifications might be involved in the ActD-mediated remodeling of chromatin conformation at the 45S rDNA regions.

### γ-H2AX accumulates at 45S rDNA sites after treatment with ActD in maize

**Figure 7:**
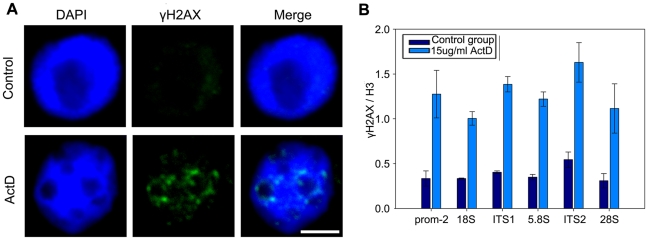
ActD treatment accumulates γH2AX within 45S rDNA regions in maize.. (A) Indirect immunofluorescence staining with an antibody against γH2AX showed weak staining signals surrounded the nucleolus without treatment but intense staining signals surrounded fragmented nucleoli after treatment with 15 µg/ml ActD. Bar = 10 µm. (B) ChIP analysis of γH2AX levels within 45S rDNA regions in maize treated without or with 15 µg/ml ActD. DNA associated with γH2AX was immunoprecipitated with the anti-γH2AX antibody and primers specific for different regions of 45S rDNA were used to amplify DNA for quantitative real-time PCR. The y-axis values were the relative quantities of γH2AX levels relative to total H3 at each amplicon and the x-axis indicated different regions of 45S rDNA. Each experiment was repeated three times and the average value was shown with the SD.

γ-H2AX is a phosphorylated histone H2A variant and serves as a hallmark of DNA double-strand breaks (DSBs) [33]. Therefore, we detected the distribution of γ-H2AX at 45S rDNA regions. The results showed that γ-H2AX signals were intensely distributed around fragmented nucleoli after ActD treatment, whereas the normal nucleoli appeared to contain only weak signals (Figure 7A). Furthermore, ChIP analysis also revealed a significant increase of γ-H2AX within the 45S rDNA regions (Figure 7B). The accumulation of γ-H2AX in ActD-treated samples indicated the presence of DNA breaks across the highly decondensed 45S rDNA chromatins, consistent with a previous conclusion that chromosome fragile sites are preferred regions for DSB formation when the DNA replication process was partially inhibited [34].

## Discussion

Here we demonstrate that the plant 45S rDNA repeats are transcription-dependent as well as replication-dependent fragile sites whose phenotypes resemble those of human chromosome fragile sites and establish that the appearance of 45S rDNA fragile sites are associated with epigenetic alterations and DNA damage.

The 45S rDNA arrays have been early defined as the secondary constriction and this concept prohibits us from connecting occasionally-observed constrictions or abnormally decondensed rDNA chromatins to fragile phenotypes. The actual underlying structural organization is completely different between the primary constriction centromeres and the secondary constriction NORs [35], although both of them display the constriction phenotype, which is also one of cytological hallmarks of fragile sites [36]. Moreover, the 45S rDNA fragile sites display not only constriction phenotypes but also gaps or breaks with no or a few thin DNA fibers observed upon routine cytological slide preparation from some plants [8], [37], [38]. In this study, highly varied fragile phenotypes were observed ranging from spatially separated breakage to highly stretched strands of 45S rDNA signals in the presence of APH, resembling the cytological appearances of human fragile sites, whereas the centromeres and knobs were not affected by treatment with APH. Therefore, we conclude that plant rRNA gene clusters are replication-associated chromosome fragile sites. Similarly, telomeres were recognized recently as fragile sites by FISH [39].

Fragile sites in human cells have been well characterized and fragile phenotypes are generally due to DNA replication-dependent defects. Specifically, site-specific gaps or breaks in chromosomes can be induced and enhanced following partial inhibition of DNA synthesis by treatment with the DNA polymerase inhibitor APH [40]. High transcriptional activity was also shown to retard local chromatin condensation, especially for tandemly repeated genes [41]. Moreover, previous results have proposed that adenovirus type 12 induces chromosomal fragile sites by disturbing the balance between transcription and condensation in human cells [17]. The results of our Ag-NOR staining experiments suggested that highly efficient transcription might contribute to the spontaneous formation of 45S rDNA lesions in *Lolium*. Actually, approximately half of rDNA repeats are transcriptionally silent and restricted to a condensed state in normal cells [42]. The occurrence of a secondary constriction may be due to local active transcription which interferes with chromosomal condensation [43]. It has been demonstrated that 5,6-dichloro-β-D- ribofuranosylbenzimidazole (DRB)-induced transcription inhibition could disturb the well-organized structure and induce highly decondensed necklace-like rRNA genes throughout human interphase nuclei [44]. In this study, a general transcription inhibitor (ActD) induced 45S rDNA decondensed phenotypes in both metaphase chromosomes and interphase nuclei in all treated plants. ActD also induced *RNU1* and *RNU2* fragility in human cells [41]. Futher rRNA expression analysis showed that ActD-induced transcriptional stress caused more rRNA genes to escape from their specific topological constraints and become accessible, which is agreement with the reported results [27]. It might be postulated that the ActD-induced active transcription units hinder rDNA chromatin condensation, ultimately resulting in the formation of the 45S rDNA fragile site.

Further analysis demonstrated that the ActD-induced 45S rDNA fragile phenotypes are associated with decreased DNA methylation, decreased levels of histone H3, and increased histone acetylation and levels of H3K4me2. Posttranslational modification of histones dynamically alters chromatin conformation and regulates the transition from interphase chromatin to highly condensed metaphase chromosomes during mitosis [45], [46]. A mutually reinforcing cross-talk between histone modifications and DNA methylation is involved in cell cycle progression in both plants and mammals [47], [48]. Our results suggest that the epigenetic alterations at 45S rDNAs are indicators of the highly decondensed states that may prevent chromatin fibers from folding into higher-order metaphase chromosomes, leading to detectable chromosome lesions at the 45S rDNAs. Alternatively, as histone acetylation has been demonstrated to correlate with the replication timing in S phase [49], the ActD-induced epigenetic changes in rDNA chromatin may retard the DNA replication process, ultimately resulting in local chromatin-packing defects. In addition, ActD binding may prevent the religation step of topoisomerase I, resulting in the accumulation of DSBs particularly focused on the sites where a replication fork encountered the stalled topoisomerase [50], [51]. Thus, a third possibility is that the ActD-induced DSBs block chromatin compaction, a model that is consistent with previous work demonstrating that DNA damage interferes with local chromatin packaging and promotes the formation of open, relaxed chromatin domains [52], [53]. The significantly accumulated γ-H2AX is a good reason to support this model. In conclusion, we define a transcription-dependent fragile expression process for 45S rDNA sites and this fragility may be associated with the DNA damage signaling pathway.

The plant rRNA gene is a tandemly repetitive cluster and its coding regions are highly conserved among species because the 45S rDNA repeats play multifunctional and important roles in eukaryotes. However, the highly repetitive nature makes them as ideal substrates for homologous recombination, so the size, distribution and number of 45S rDNA are much variable among different plants [54], [55] reflecting phylogenetic distances between species and genera. Fragile rDNA phenotypes as constrictions, gaps or breaks in metaphase chromosomes also suggest an aberrance in chromosome organization. Thus fragility of 45S rDNA sites appears to be a driving force for genome instability and evolution of genomic architecture [56]. The numbers and positions of the rDNA sites widely varied among different accessions in *Lolium* and no two plants had the same pattern of rDNA sites, even within the same root meristem [57]. The reduction of 45S rDNA loci occurred in wheat genome evolution [58]. A tetraploid species in *Gossypium* had many minor rDNA sites which were not detected in the diploid ancestral species [59]. Thus, the rRNA gene is an important marker used in constructing phylogenetic relationships among different plants and further comprehending plant evolutionary history [60]. The variation in size and number of 45S rDNA sites could reflect 45S rDNA fragility that often expressed breaking and subsequent rejoining of chromosomes within 45S rDNA sites. The fragile nature makes them serve as hot spots for rearrangements originating from intra- and inter-strand recombination and preferred sites for foreign gene integration, both of which seem to have an effect on the varied copies and distribution [61]. As 45S rDNA is one of the most fragile sites in the genome, eukaryotic cells evolve a very complex amplification system to maintain its stability. Recent advance proposes that extra 45S rDNA copies possess the ability to maintain genome integrity by repressing transcription-dependent defects and facilitate recombination repair by accumulating condensin and cohesion association [62], [63].

## Materials and Methods

### Plant materials and treatments

Seeds of the *Lolium perenne* L. diploid turf type cultivar ‘Player’ (2n = 14) were kindly provided by Turf Seed (Hubbard, OR, USA). Seeds of the *Zea mays* L. inbred line ‘Nongda 108’ (2n = 20) were obtained from China Agricultural University (Beijing, China). Seeds of the *Oryza sativa* L. indica ‘9311’ (2n = 24) and *Hordeum vulgare* L. cv. ‘Plaisant’ (2n = 14) were obtained from State Key Laboratory of Hybrid Rice of Wuhan University (Wuhan, China). Seeds of the *Sorghum bicolor* L. ‘Moench’ (2n = 20) were provided by Yangzhou University (Yangzhou, China). Seeds were germinated at 25°C on cotton gauzes soaked in water. Seedlings were treated for 48 h at 25°C with or without varying concentrations of APH (Wako Chemicals USA, Inc., Richmond, VA, USA) or ActD (Amersco, SF, USA) when the primary roots were approximately 1.0 cm long. 15 µg/ml and 50 µg/ml APH and 5 µg/ml and 15 µg/ml ActD were selected for current research.

### Chromosome preparation and nucleus isolation

The procedure for metaphase chromosome preparation was adopted from Li et al. [64]. To block mitosis at metaphase, ryegrass and barley roots were treated in ice cold water for 24 h; maize and sorghum roots were pre-treated with α-bromonaphthalene for 4 h; and rice roots were not pretreated. After being pre-treated, all the roots were fixed in Carnoy's fixative [absolute ethanol: acetic acid (3∶1, v/v)] for at least 24 h. Then, roots were digested with a mixture (2% pectolyase and 2% cellulase) for varied hours (1 h for ryegrass, 1.5 h for barley, rice and sorghum, 2 h for maize) at 37°C. Finally, the enzyme-digested root tips were crushed onto slides and air dried.

Pure nuclei were isolated using the method proposed by Li et al. [65]. An appropriate amount of root tips were chopped in extraction buffer (0.01 M MgSO_4_, 5 mM KCl, 0.5 mM Hepes, 1 mg/ml dithiothreitol, and 0.25% Triton X-100, pH 7.0) and filtered through a 33 µm nylon mesh filter. The nuclei were centrifuged at 200 g for 10 min at 4°C and resuspended in the same buffer. Then, nuclei were fixed in 4% paraformaldehyde in 1×PBS for 1 h at the room temperature and spread on slides.

### AgNOR-staining

The selective staining of Ag-NOR proteins was carried out according to the protocol proposed by Howell and Black [66]. Chromosome preparations were covered with a mixing staining solution in proportions of 2∶1 (v/v) [0.5 g/ml AgNO_3_ in deionized water: 0.02 g/ml gelatin in 1% (v/v) formic acid] and incubated for 2–5 min at 72°C until the staining mixture turned golden-brown. After staining, the slides were washed with running deionized water and dehydrated in an ethanol series. Images were then captured using a light microscope. Subsequently, the selected slides were subjected to the FISH experiment after washing in 2×SSC and ethanol series.

### Fluorescence in situ hybridization (FISH) and immuno-staining assay

FISH was performed as previously described [67]. The slides were treated in 70% formamide in 2×SSC under different conditions (2.5 min at 72°C for carnoy-fixed slides and 5 min at 90°C for paraformaldehyde-treated slides). Simultaneously, 1–2 µg/ml probes and 1 mg/ml sheared salmon sperm DNA were pre-mixed in 2×SSC with 10% dextran sulphate and 50% deionized formamide, and denatured at 75°C for 5 min before cooled with ice/water immediately. After dehydrating and air drying, the slides were incubated in denatured hybridization solution for overnight at 37°C. Biotin-labeled probes were then detected and amplified with streptavidin-Cy3 (Jackson Immunoresearch, West Grove, PA) and biotinylated anti-streptavidin antibody (Vector Laboratories, Burlingame, CA) respectively. Digoxigenin-labeled probes were detected and amplified with sheep-anti-digoxin-FITC (Roche, Lewes, UK) and rabbit-anti-sheep-FITC (Roche, Lewes, UK) respectively. Each immune reaction was performed for 1 h at 37°C before washing with 1×PBS. Nuclei and chromosomes were stained with 4′, 6-diamidino-2-phenylindole (DAPI, 0.2 µg/ml, Sigma, Deisenhofen, Germany) and observed under an Olympus BX-60 fluorescence microscope with appropriate filters for DAPI, Cy3 and FITC respectively. Images obtained using a CCD monochrome camera Sensys 1401E were pseudo-colored and processed with Metamorph imaging system (Universal Imaging Corp., PA, USA. version 4.6.3) and Adobe Photoshop 9.0 software.

The immunostaining assay was adopted to detect the distribution of fibrillarin and γ-H2AX. Paraformaldehyde-fixed nuclei were blocked with 3% bovine serum albumin (BSA, Sigma, Deisenhofen, Germany) dissolved in 1×PBS at 37°C for 1 h before washed with 1×PBS for three times, each for 3 min. Then, the slides were incubated with anti-fibrillarin antibody (Abcam, Cambridge, UK. # ab4566) or anti-γ-H2AX antibody (Abcam, Cambridge, UK. # ab2893) for overnight at 4°C. The antibodies were diluted with 3% BSA in proportions of 1∶100. After washing with 1×PBS for 5 min, the slides were incubated at 37°C for 1 h with secondary antibodies [fluorescein-conjugated goat anti-mouse IgG for fibrillarin (Millipore, MA, USA. # 401214-2ML), fluorescein-conjugated goat anti-rabbit IgG for γ-H2AX (Millipore, MA, USA. # 401314-2ML). Immunostained slides were then washed in 1×PBS and counterstained with DAPI. As mentioned above, images were captured and processed.

### Quantitative real-time PCR assay

Total RNA was isolated using the RNAprep pure Plant Kit (Qiagen, Germany) and contaminating genomic DNA was removed by DNase I treatment (Fermentas, Canada) followed by phenol∶chloroform∶isoamyl alcohol (25∶24∶1) extraction. The purified RNA was reverse-transcribed to cDNA by using RevertAid First Strand cDNA Synthesis Kit (Fermentas, Canada). Quantitative real-time PCR was performed using a StepOne Plus real-time PCR system (Applied Biosystems, Carlsbad, USA) in the presence of SYBR® Green Real-time PCR Master Mix (TOYOBO, Tokyo, Japan). The amplification parameters were as follow: 94°C for 2 min, followed by 40 cycles at 94°C for 5 sec, 58°C for 15 sec, and 72°C for 25 sec. Fluorescence data were acquired at the 72°C step and during the melting-curve program. Preliminary experiments were run to ensure the amplification of a single PCR product for each gene. Quantitative real-time PCR were repeated three times for each sample from three independent experiments.

### DNA methylation analysis

Genomic DNA was isolated and modified with sodium bisulfite as described [30], [68]. The primers specific for the 45S rDNA promoter region (prom-1) from −133 to +174 were designed by MethPrimer software (Table 1). Amplified products were purified using a gel extraction kit (Qiagen, Mannheim, Germany) and transformed into DH5α E. coli cells using pGem-T easy (Promega, Madison, WI, USA). Twenty clones were sequenced for each sample and analyzed with BiQ Analyzer software.

### Chromatin immunoprecipitation (ChIP)

ChIP assays were performed using standard procedures [69]. Equal amounts of chromatin extracts were digested into 200–500 bp with micrococcal nuclease and then incubated overnight at 4°C with histone related antibodies including anti-Histone H3 (Millipore, MA, USA. #06-755), anti-H3K9ac (Millipore, MA, USA. #07-352), anti-H3K9me2 (Millipore, MA, USA. #17-648), anti-H3K4me2 (Millipore, MA, USA. #07-030), anti-H4K5ac (Millipore, MA, USA. #07-327) and anti- H4K16ac (Millipore, MA, USA. #17-10101). Immunoprecipitation using rabbit serum was performed as a negative control. Following ChIP, Quantitative real-time PCR were performed with the primers specific for the promoter (prom-2), 18S, ITS1, 5.8S, ITS2 and 28S (Table 1).

## Supporting Information

Figure S1 **Effects of APH on centromeres and knobs in maize.** (A) FISH mapping on metaphase chromosomes combined centromere probes (red) with 45S rDNA probes (green) showed that APH induced 45S rDNA lesions but had no effect on centromeres in the same metaphase spread after treatment with 15 µg/ml APH. Bar = 5 µm. (B) FISH image showed that decondensation of 45S rDNA was observed, but all of the centromeric sites remained intact blocks of heterochromatin in the same nucleus after treatment with 15 µg/ml APH. Bar = 10 µm. (C) FISH mapping combined knob probes (red) with 45S rDNA probes (green) showed that APH (15 µg/ml) induced NOR lesions but no visible damage on the knob regions in the same metaphase spread. Bar = 5 µm. (D) FISH image showed that extensive decondensation of 45S rDNA was observed, but all of the knob regions remained intact blocks of heterochromatin in the same nucleus after treatment with 15 µg/ml APH. Bar = 10 µm.
(TIF)

Figure S2 **Effects of ActD on centromeres and knobs in maize.** (**A**) FISH image showed that all of the centromeric sites remained intact blocks of heterochromatin in both metaphase chromosome spread and interphase nucleus after treatment with 15 µg/ml ActD. Bar = 10 µm. (B) FISH image showed that all of the knob regions remained dense spots in both metaphase chromosome spread and interphase nucleus after treatment with 15 µg/ml ActD. Bar = 10 µm.
(TIF)
