# Supplementary figures and images for: Plant 45S rDNA Clusters Are Fragile Sites and Their Instability Is Associated with Epigenetic Alterations
Source: PLoS One. 2012 Apr 11;7(4):e35139. doi: 10.1371/journal.pone.0035139 (PMC3324429; doi:10.1371/journal.pone.0035139)

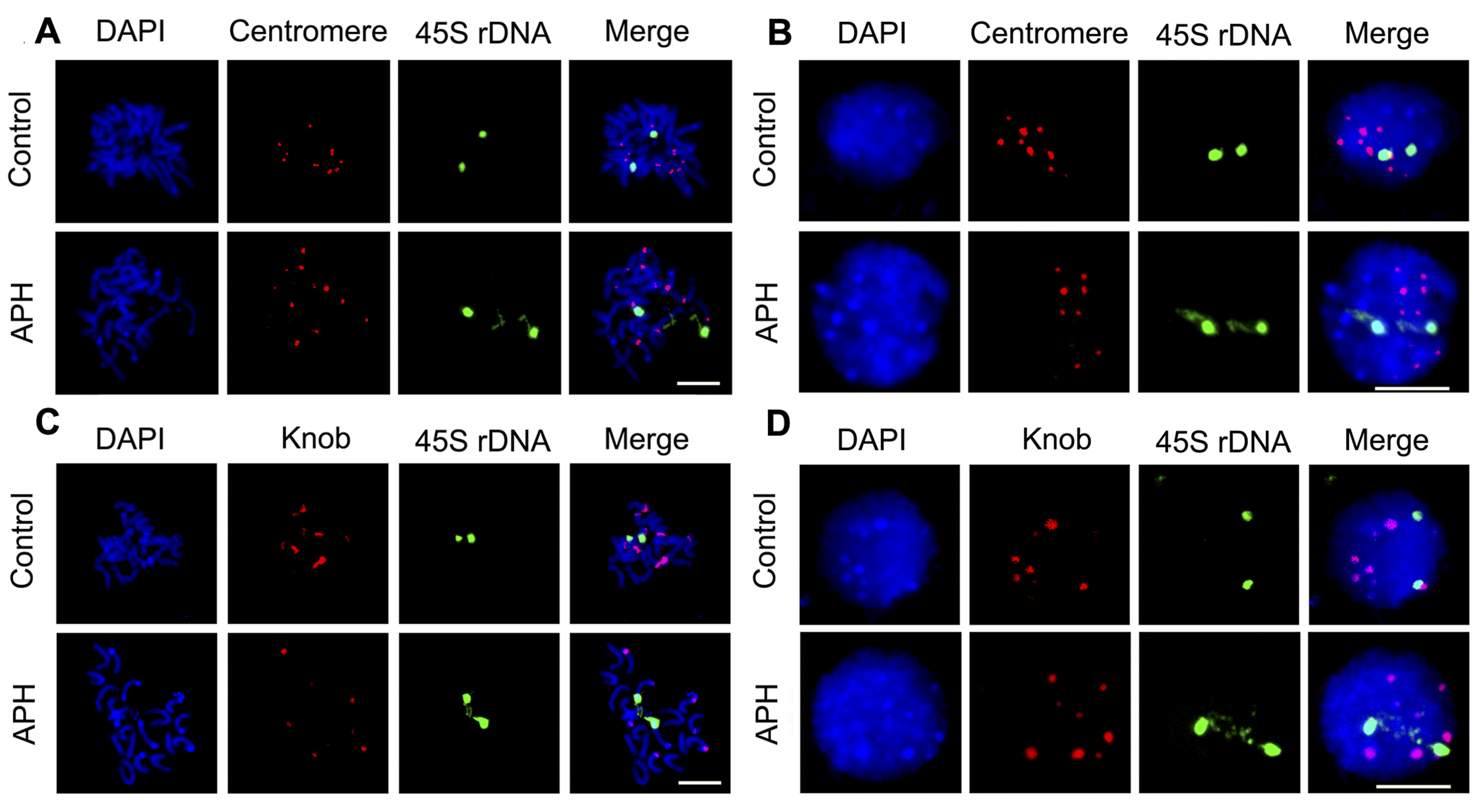

Supplement: Figure S1 — Effects of APH on centromeres and knobs in maize. (A) FISH mapping on metaphase chromosomes combined centromere probes (red) with 45S rDNA probes (green) showed that APH induced 45S rDNA lesions but had no effect on centromeres in the same metaphase spread after treatment with 15 µg/ml APH. Bar = 5 µm. (B) FISH image showed that decondensation of 45S rDNA was observed, but all of the centromeric sites remained intact blocks of heterochromatin in the same nucleus after treatment with 15 µg/ml APH. Bar = 10 µm. (C) FISH mapping combined knob probes (red) with 45S rDNA probes (green) showed that APH (15 µg/ml) induced NOR lesions but no visible damage on the knob regions in the same metaphase spread. Bar = 5 µm. (D) FISH image showed that extensive decondensation of 45S rDNA was observed, but all of the knob regions remained intact blocks of heterochromatin in the same nucleus after treatment with 15 µg/ml APH. Bar = 10 µm. (TIF) [file pone.0035139.s001.tif]

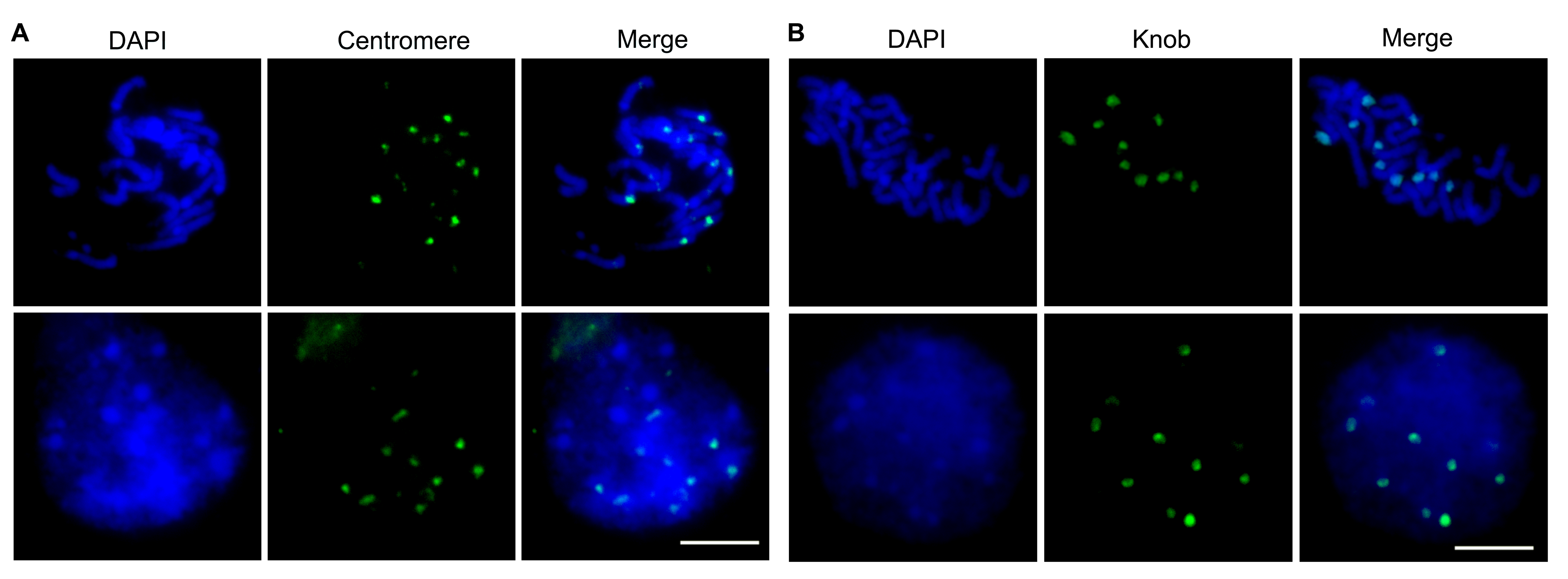

Supplement: Figure S2 — Effects of ActD on centromeres and knobs in maize. (A) FISH image showed that all of the centromeric sites remained intact blocks of heterochromatin in both metaphase chromosome spread and interphase nucleus after treatment with 15 µg/ml ActD. Bar = 10 µm. (B) FISH image showed that all of the knob regions remained dense spots in both metaphase chromosome spread and interphase nucleus after treatment with 15 µg/ml ActD. Bar = 10 µm. (TIF) [file pone.0035139.s002.tif]
